# Oxidative stress induces BH_4_ deficiency in male, but not female, SHR

**DOI:** 10.1042/BSR20180111

**Published:** 2018-07-03

**Authors:** Ellen E. Gillis, Krystal N. Brinson, Olga Rafikova, Wei Chen, Jacqueline B. Musall, David G. Harrison, Jennifer C. Sullivan

**Affiliations:** 1 Department of Physiology, Augusta University, Augusta, GA, U.S.A.; 2 Department of Medicine, University of Arizona, Tucson, AZ, U.S.A.; 3 Division of Clinical Pharmacology, Department of Medicine, Vanderbilt University, Nashville, TN, U.S.A.

**Keywords:** hypertension, kidney, nitric oxide, sex

## Abstract

We previously published that female spontaneously hypertensive rats (SHR) have significantly greater nitric oxide (NO) bioavailability and NO synthase (NOS) enzymatic activity in the renal inner medulla (IM) compared with age-matched males, although the mechanism responsible remains unknown. Tetrahydrobiopterin (BH_4_) is a critical cofactor required for NO generation, and decreases in BH_4_ as a result of increases in oxidative stress have been implicated in the pathogenesis of hypertension. As male SHR are known to have higher levels of oxidative stress compared with female SHR, we hypothesized that relative BH_4_ deficiency induced by oxidative stress in male SHR results in lower levels of NOS activity in renal IM compared with females. Twelve-week-old male and female SHR were randomized to receive tempol (30 mg/kg/day via drinking water) or vehicle for 2 weeks. Tempol treatment did not affect blood pressure (BP) in either sex, but reduced peroxynitrite levels only in males. Females had more total biopterin, dihydrobiopterin (BH_2_), and BH_4_ levels in renal IMs than males, and tempol treatment eliminated these sex differences. Females had greater total NOS activity in the renal IM than males, and adding exogenous BH_4_ to the assay increased NOS activity in both sexes. This sex difference in total NOS and the effect of exogenous BH_4_ were abolished with tempol treatment. We conclude that higher oxidative stress in male SHR results in a relative deficiency of BH_4_ compared with females, resulting in diminished renal NOS activity in the male.

## Introduction

The nitric oxide (NO)/NO synthase (NOS) pathway is critical in blood pressure (BP) regulation [1–3]. Deficiencies in NO are correlated with the incidence and progression of hypertension [1,4–6] and in particular, renal NOS has been shown to be important in modulating BP [7]. The renal inner medulla (IM) has the highest amount of NOS protein expression and enzymatic activity in the kidney [8] and NO regulates inner medullary blood flow and inhibits transport of sodium chloride along the nephron [9,10]. Moreover, both clinical and experimental studies have documented greater NO production and bioavailability in females compared with males. Previously, we published that the renal IM is the only section of the kidney to exhibit sex differences in NOS enzymatic activity in young adult (13 weeks old) spontaneously hypertensive rats (SHR) with greater total NOS enzymatic activity in female SHR compared with males [11–16]. We further showed that female SHR exhibit a sex hormone‐ and BP‐dependent increase in NOS activity with maturation that is not observed in males [15], although why NOS activity does not increase with maturation in males remains unknown. Elucidating the molecular mechanism(s) driving the sexual dimorphism in renal IM NOS activity may provide insight into sex differences in not only the NO action in the kidney, but also BP regulation.

NOS catalyzes the formation of NO from l-arginine and oxygen in a reaction that requires a number of cofactors, including tetrahydrobiopterin (BH_4_). In the absence of BH_4_, electron flow to molecular oxygen is ‘uncoupled’ from l-arginine oxidation and NO formation instead resulting in the production of superoxide. In addition to decreasing NO production, superoxide is highly reactive with NO, resulting in the formation of peroxynitrite. Peroxynitrite rapidly oxidizes BH_4_ to dihydrobiopterin (BH_2_), and as BH_2_ is not a NOS cofactor, it can competitively inhibit the binding of BH_4_ to NOS [17,18]. As a result, NOS uncoupling serves as both a cause and effect of BH_4_ deficiencies. Furthermore, decreases in BH_4_ and uncoupled NOS have been implicated in numerous cardiovascular diseases, including hypertension [19,20].

Based on the finding that male SHR have greater levels of oxidative stress compared with female SHR [21,22], coupled with lower levels of NOS activity [15,16], the goal of the current study was to test the hypothesis that relative oxidative stress-induced BH_4_ deficiency in male SHR is responsible for lower levels of NOS activity in renal IM compared with females.

## Experimental

### Animals

Twelve-week-old male and female SHR (Envigo, Inc., Indianapolis, Indiana) were studied. All experiments were conducted in accordance with the National Institutes of Health *Guide for the Care and Use of Laboratory Animals* and approved and monitored by the Augusta University Institutional Animal Care and Use Committee. Male and female SHR were randomized to receive tempol, a reactive oxygen species scavenger (30 mg/kg/day; Sigma–Aldrich; *n*=8–9) or vehicle (tap water; *n*=11–12) in their drinking water for 2 weeks. Water intake was measured daily and rats were weighed every 3 days to maintain appropriate dosing throughout the study. A separate set of male and female SHR were implanted with telemetry devices for the continuous measurement of BP (*n*=5). Rats were implanted with telemetry devices at 10 weeks of age as previously described [23], allowing 1 week of recovery and 1 week of baseline BP recording before initiating treatment with tempol. An additional set of 12-week-old male SHR were randomized to receive BH_4_ supplementation (20 mg/kg/day; Axxora, LLC, San Diego, CA) or vehicle (saline) via daily ip injection for 1 week (*n*=8/group). Systolic BP was measured in all rats prior to initiating treatment and following 7 days of treatment via tail-cuff plethsomyography as previously described [24]. For all studies, rats were anesthetized with ketamine/xylazine (50 mg/kg/10 mg/kg, i.p.), and a terminal blood sample was taken and centrifuged for collection of plasma. Kidneys were removed and the renal IM were isolated and snap-frozen in liquid nitrogen.

### Dot blot analysis

Plasma samples were diluted and 1.5 µl diluted plasma was applied to a nitrocellulose membrane forming a dot and allowed to dry overnight. Membranes were then blocked with 1% BSA in TBS, 0.1% Tween 20 (TBST) for 1 h and incubated with anti-3-nitrotyrosine (3-NT) antibody at a final concentration of 1 mg/ml (Calbiochem) at room temperature for 2 h. The membrane was washed with TBST, incubated with secondary antibody, washed again, and imaged using an Odyssey Imaging System (LI-COR Biosciences, Lincoln, NE). To quantitate the total protein in each sample, the membrane was incubated with Ponceau S BioReagent for 15 min. The data were presented as a ratio of 3-NT signal per total protein signal.

### NOS enzymatic activity assay

The renal IMs were homogenized as previously described [25] and the whole homogenate was then used in the NOS activity assay in the presence or absence of exogenous 3 µM BH_4_ as previously described [16]. Briefly, total NOS activity was determined based on the rate of l-[^3^H]citrulline formation from l-[^3^H]arginine and defined as [^3^H]arginine to [^3^H]citrulline conversion inhibited by the nonselective NOS inhibitor *N*-nitro-l-arginine (l-NNA; 1 mmol/l).

### Biopterin analysis

Biopterins were measured by HPLC. Briefly, renal IMs were homogenized in 300 μl ice-cold Lysis buffer (50 mM Tris/HCl, pH 7.4, 1 mM DTT, 1 mM EDTA). Two hundred seventy microliters of extract was added to 30 μl of a 1:1 mixture of 1.5 M HCLO_4_ and 2 M H_3_PO_4_. Concentrations of BH_4_ and BH_2_ were determined using HPLC and differential oxidation as previously described [26]. Samples were then centrifuged for 10 min at 14000 ***g*** at 4°C, and the resulting precipitate was re-suspended in 100 μl of 1 M NaOH and analyzed for total protein content (Bradford assay) as previously described [26].

### Western blot analysis

Renal IMs were homogenized as previously described [25] and the whole homogenate was used in Western blot analysis as previously described [16]. Briefly, protein expression was determined using two-color immunoblots using primary antibodies to GTP cyclohydrolase-1 (GTPCH1; 1:250, Santa Cruz Biotechnology, Santa Cruz, CA, 100 μg protein/well) and β-actin (A1978, 1:10000; Sigma, St. Louis, MO). Protein concentrations were determined by standard Bradford assay (Bio-Rad, Hercules, CA) using BSA as the standard. β-actin was used to verify equal protein loading, and data were reported normalized to β-actin.

### Statistical analysis

All data are expressed as means ± S.E.M. BP was analyzed using repeated-measures ANOVA to examine within-group effects and Student’s *t*test to examine between-group effects. Biopterin levels between control and BH_4_ treated male SHR were analyzed using Student’s *t* test. All other data were compared using a two-way ANOVA followed by a Newman–Keul’s post-hoc. For all comparisons, *P*<0.05 was considered statistically significant. Analyses were performed using GraphPad Prism version 7.0 software (GraphPad Software Inc, La Jolla, CA).

## Results

### Tempol decreases measures of oxidative stress independent of BP

**Figure 1:**
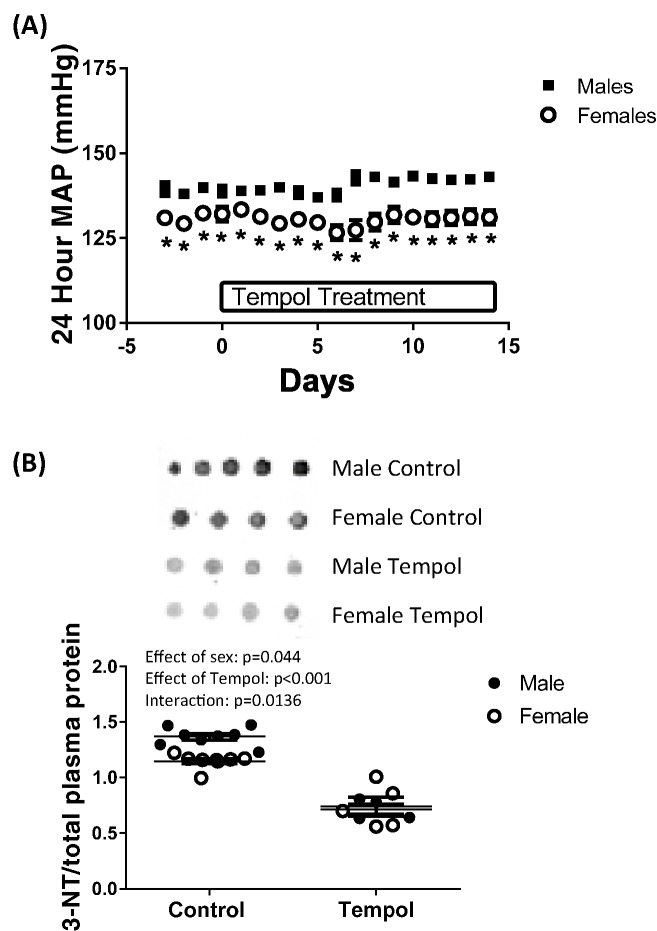
Tempol decreases measures of oxidative stress independent of BP (**A**) Mean arterial pressure (MAP) was measured by radiotelemetry in male and female SHR receiving tempol (30 mg/kg/day) in drinking water, *n*=5. Data are presented as mean ± S.E.M., *P<0.05 compared with male. (**B**) Plasma levels of 3-NT were measured in vehicle control (*n*=5) and tempol-treated (*n*=8) male and female SHR by Dot blot analysis.

BP was measured by telemetry in male and female SHR treated with tempol; Figure 1A. Male SHR had a higher BP compared with female SHR at baseline (140 ± 2 compared with 132 ± 2; *P*=0.013). BP remained higher in males throughout the treatment period, although tempol had no effect on BP in either sex (effect of tempol in males, *P*=0.25; effect of tempol in females, *P*=0.096).

Peroxynitrite is formed by the binding of NO and superoxide, and BH_4_ is a target for oxidation by peroxynitrite leading to uncoupled NOS [17,18]. Peroxynitrite reacts with tyrosine residues in proteins resulting in the formation of 3-NT [27,28]. Therefore, plasma 3-NT levels were measured in vehicle and tempol-treated male and female SHR using Dot blot analysis; Figure 1B. Male SHR had greater levels of 3-NT than females (effect of sex, *P*=0.044; Figure 1B). Treatment with tempol reduced 3-NT levels only in males, abolishing the sex difference (effect of tempol, *P*<0.0001; interaction, *P*=0.014).

### Tempol treatment abolishes the sex difference in IM NOS activity and dependency on exogenous BH_4_

**Figure 2:**
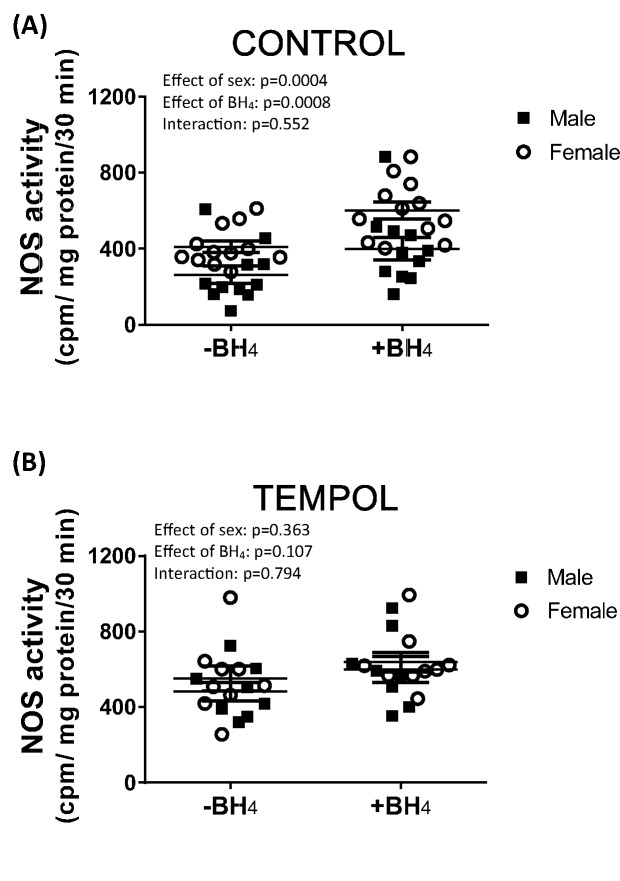
Tempol treatment abolishes the sex difference in IM NOS activity and dependency on exogenous BH_4_ Total NOS enzymatic activity in the renal IM of vehicle control ((**A**); *n*=11–12) and tempol-treated ((**B**); *n*=8–9) male and female SHR (*n*=8–12) with and without the addition of exogenous BH_4_.

The measurement of NOS enzymatic activity via detection of the formation of radiolabeled citrulline (and NO) from arginine is typically performed in the presence of excess amounts of all NOS cofactors, including BH_4_. In the current study, we measured NOS enzymatic activity in the renal IM of vehicle control and tempol-treated male and female SHR in the absence and presence of exogenous BH_4_; Figure 2. Consistent with our previous publications [15,16], total NOS enzymatic activity was lower in the renal IM of control, vehicle-treated male SHR compared with female SHR (effect of sex, *P*=0.0004). The inclusion of BH_4_ in the assay increased NOS activity in both sexes, and the increase was comparable in males (36 ± 5% increase) and females (30 ± 4% increase; effect of BH_4_, *P*=0.0008; interaction, *P*=0.55). In contrast, following treatment with tempol there was no sex difference in NOS activity (effect of sex, *P*=0.36) and inclusion of exogenous BH_4_ did not significantly increase NOS activity in either sex (effect of BH_4_, *P*=0.11; interaction, *P*=0.79).

### Male SHR have lower biopterin levels in the renal IM compared with female SHR

**Figure 3:**
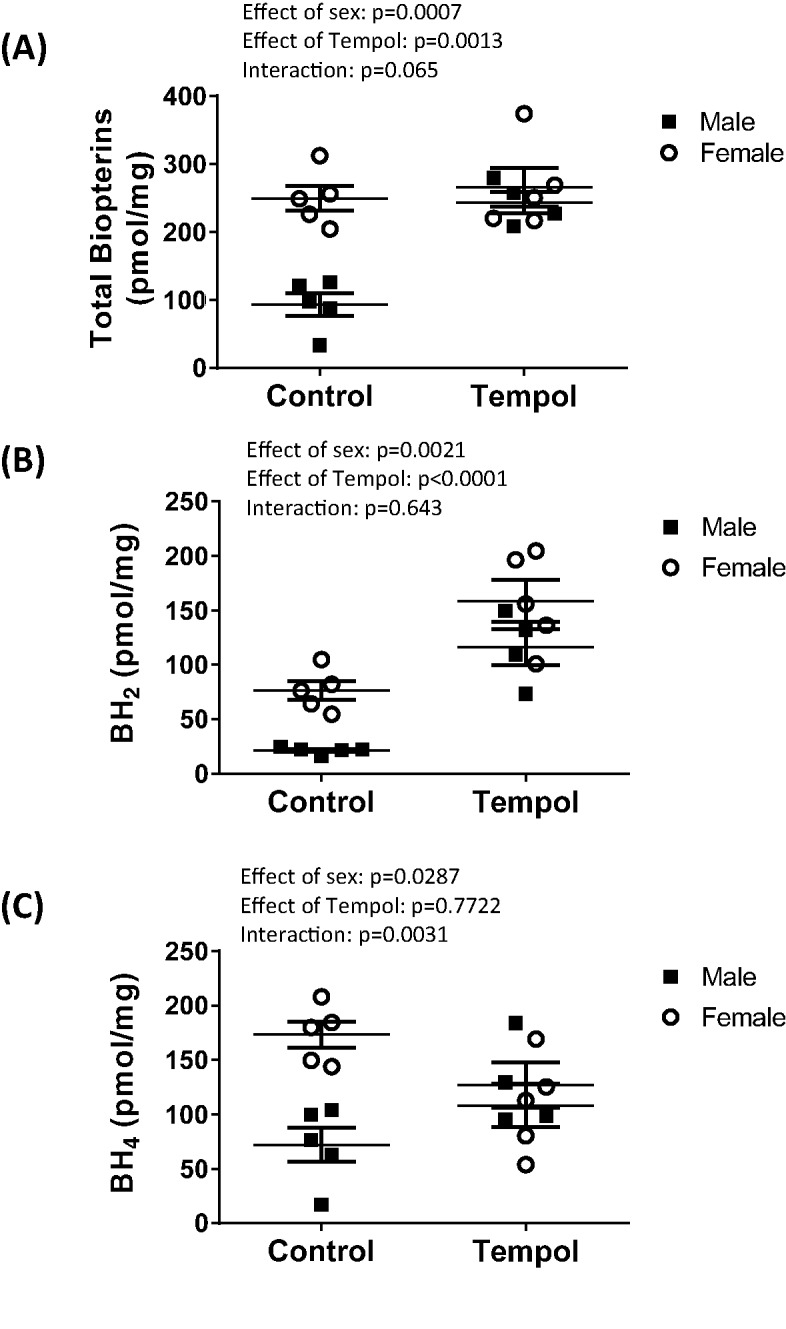
Male SHR have lower biopterin levels in the renal IM compared with female SHR HPLC analysis of total biopterins (**A**), BH_2_ (**B**), and BH_4_ levels (**C**) in renal IM of vehicle control and tempol-treated male and female SHR (*n*=4–5).

Total biopterin, BH_2_ and BH_4_ levels were quantitated in IM from vehicle control and tempol treated male and female SHR via HPLC analysis; Figure 3. Males have lower levels of total biopterins (effect of sex, *P*=0.0007), BH_2_ (effect of sex, *P*=0.0021), and BH_4_ compared with females (effect of sex, *P*=0.029). Sex differences in biopterins, BH_2_, and BH_4_ were abolished by tempol treatment. Treatment with tempol increased total biopterins only in males (effect of tempol, *P*=0.0013; interaction, *P*=0.065). BH_2_ levels were increased by tempol in both sexes (effect of tempol, *P*<0.0001; interaction, *P*=0.643), while BH_4_ levels increased only in male SHR with tempol (effect of tempol, *P*=0.7722; interaction, *P*=0.0031).

### GTP cyclohydrolase expression is comparable in male and female SHR

**Figure 4:**
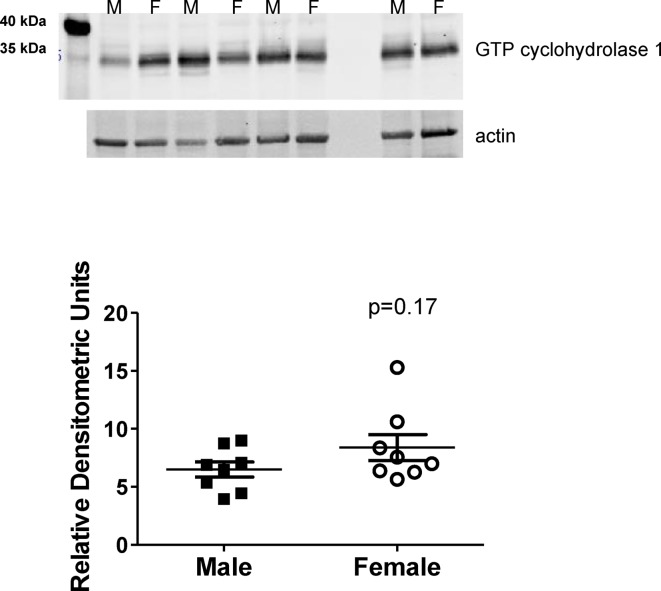
GTP cyclohydrolase expression is comparable in male and female SHR GTP cyclohydrolase 1 protein was measured by Western blot analysis in renal IM of vehicle control male and female SHR (*n*=8). Data are shown normalized to actin.

To determine if sex differences in BH_4_ levels were mediated by differences in production, GTPCH1 protein expression was measured; GTPCH1 is the rate-controlling enzyme in BH_4_ production. GTPCH1 protein expression is comparable in the renal IM of male and female SHR (Figure 4; *P*=0.17).

### Exogenous BH_4_ treatment in male SHR abolishes sex differences in biopterins

**Figure 5:**
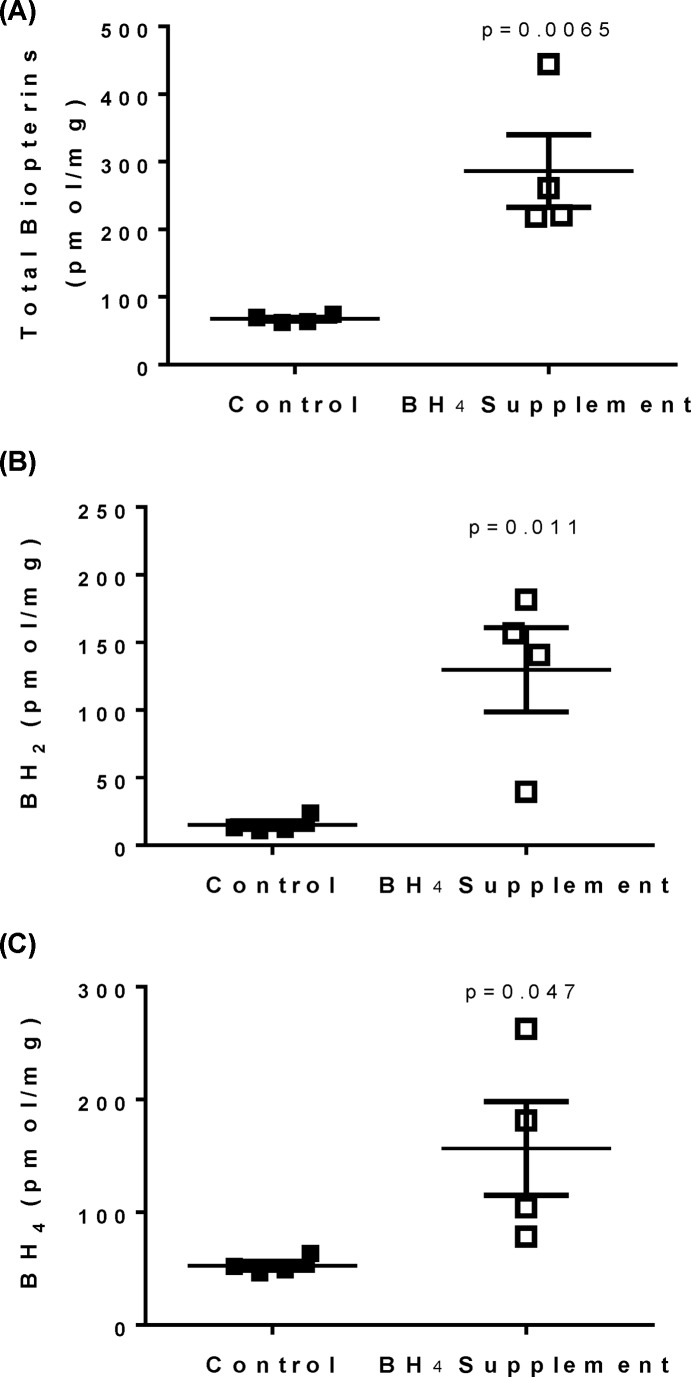
Exogenous BH_4_ treatment in male SHR abolishes sex differences in biopterins HPLC analysis of total biopterins (**A**), BH_2_ (**B**), and BH_4_ levels (**C**) in renal IM of vehicle control and BH_4_-treated male SHR (*n*=4).

**Figure 6:**
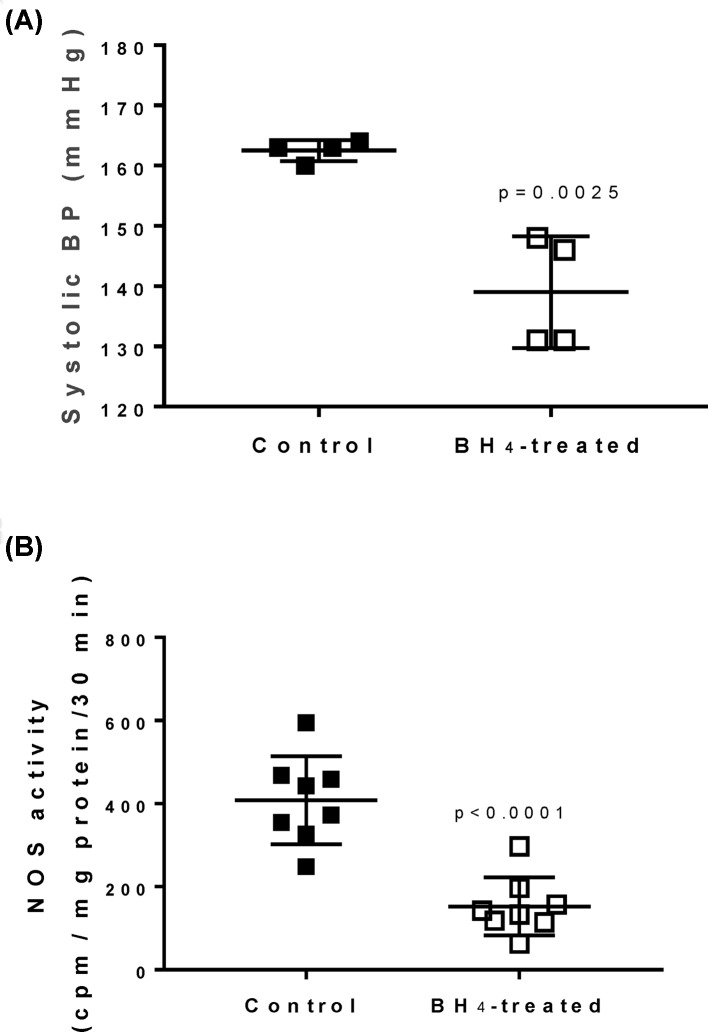
Exogenous BH_4_ treatment decreased systolic BP and NOS activity in male SHR BP, measured by tail-cuff analysis ((**A**); *n*=4) and IM NOS activity ((**B**); *n*=8) in vehicle control and BH_4_-treated male SHR.

To determine if a BH_4_ deficiency results in reduced IM NOS activity in male SHR, additional male SHR received BH_4_ supplementation for 1 week. BH_4_ supplementation increased total biotperin, BH_2_, and BH_4_ in the renal IM (Figure 5). BH_4_ supplementation of male SHR also abolished sex differences in total biotperin, BH_2_, and BH_4_ that were observed between control male and female SHR; biopterin levels in treated males and control females were compared by Student’s *t*test (total biopterin, *P*=0.38; BH_2_, *P*=0.11; BH_4_, *P*=0.86). BH_4_ treatment had no effect on body weight, although systolic BP was significantly decreased by BH_4_ supplementation (Figure 6A; *P*=0.0024).

NOS activity was then measured in the absence and presence of BH_4_ in control and BH_4_-treated male SHR. Supplementation of male SHR with BH_4_ resulted in a significant decrease in total NOS activity compared with vehicle-treated SHR (*P*<0.0001), however, the increase in NOS activity observed with the inclusion of BH_4_ in the assay was blunted (Figure 6B; *P*=0.0075).

## Discussion

Although there are numerous reports of sex differences in NO production and NO bioavailability, the molecular mechanisms responsible remain unknown. The goal of the present study was to further explore the mechanisms that result in sex differences in the NO system. Our novel hypothesis in the current study is that increased oxidative stress in male SHR induces a relative BH_4_ deficiency compared with female SHR, resulting in lower renal NOS activity. The main findings of the current study are: (i) there is an oxidative stress-induced sex difference in biopterins, where female SHR have greater biopterin levels than males and (ii) the relative deficiency in BH_4_ contributes to lower renal NOS activity in male SHR compared with female SHR. These data suggest that mechanisms to maximize BH_4_ levels will offer cardiovascular protection to hypertensive males in particular. Indeed, treating male SHR with BH_4_ significantly decreases BP, although this is not associated with an increase in total NOS activity. Additional studies are needed to fully understand the mechanisms by which BH_4_ lowers BP.

We have previously reported that there is a sex difference in NOS activity in the renal IM of SHR that cannot be explained by sex difference in NOS protein expression alone [15,16]. The relative abundance of NOS in female SHR may contribute to the lower BP observed in female SHR compared with males [6]. However, the mechanisms contributing to sex difference in NOS activity have not yet been clearly elucidated. The goal of the current study was to test the hypothesis that relative BH_4_ deficiency induced by oxidative stress in male SHR results in lower levels of NOS activity in renal IM compared with females. Male SHR have been reported numerous times to have greater levels of oxidative stress in the kidney compared with female SHR [21,25,29], although peroxynitrite in particular is known to rapidly oxidize BH_4_ to BH_2_ [17,18]. In the current study we show greater renal levels of 3-NT in male SHR compared with females, and confirmed that treatment with tempol abolishes the sex difference in 3-NT independent of an effect on BP. These findings lend support to the hypothesis that male SHR will have greater BH_4_ oxidation which may result in decrease in NOS activity. Based on the central role of the NO/NOS pathway in modulating BP and vascular tone, sex differences in biopterin levels may significantly contribute to sex differences in overall cardiovascular health.

BH_4_ is an essential cofactor for NO production. BH_4_ levels are decreased in many models of cardiovascular disease [30] including hypertension [31]. A role for BH_4_ in BP control is further supported by the finding that BH_4_ supplementation lowers BP in a male rat model of pulmonary arterial hypertension [32] and male mice with disrupted BH_4_ synthesis are hypertensive [33]. Despite growing interest and evidence for the role of BH_4_ in the pathogenesis of hypertension, there is a scarcity of data in the literature examining the impact of sex on the BH_4_ system. We report that total biopterin, BH_2_, and BH_4_ levels are all greater in females compared with males. These data support the hypothesis that there is an impaired biopterin system in the renal IM of male SHR. This hypothesis is supported by findings of lower biopterin and BH_4_ content in cardiac and vascular tissue in male SHR compared with normotensive male WKY [34,35].

NOS enzymatic activity is typically measured in the presence of excess amounts of BH_4_. However, to determine if NOS enzymatic activity is dependent on exogenous BH_4_ or if sufficient amounts are present in the tissue homogenate, we performed the NOS activity assay in the presence and absence of excess BH_4_. Despite sex differences in BP, NOS activity, and oxidative stress, vehicle control male and female SHR exhibited a comparable increase in NOS activity with the inclusion of excess BH_4_. However, treatment with tempol abolished the dependency of the NOS activity assay on excess BH_4_ in both sexes, suggesting that oxidative stress is a greater determinant of dependency on BH_4_ than sex of the animal. It should be noted that we confirmed that tempol treatment did not alter NOS protein expression (data not shown).

BH_4_ bioavailability is determined by: (i) the activity of GTPCH1, the rate-controlling enzyme in BH_4_ production and (ii) oxidative stress, which can oxidize BH_4_ to BH_2_, resulting in NOS uncoupling and the production of reactive oxygen species [36]. We found no differences in GTPCH1 protein expression in the renal IM of male and female SHR. Therefore it is unlikely that the relative deficiency in BH_4_ in male SHR is due to alterations in BH_4_ production compared with the female SHR. Activity of GTPCH1 was not assessed in the current study, therefore it is difficult to completely rule out a sex difference in production. Instead, our data support the hypothesis that oxidative stress is an important determinant of BH_4_ availability in male SHR, as treatment with tempol significantly increases BH_4_ levels in males, while BH_4_ levels in the female SHR treated with tempol do not change. However, without direct measurement of the rate of synthesis of BH_4_ in male compared with female IM lysates it is not possible to definitively demonstrate that oxidative destruction of BH_4_ is the major mechanism responsible for sex-dependent differences in BH_4_ concentrations, NOS activity, and BP in SHR.

Interestingly, BH_2_ levels are also greater in females compared with males, however the ratio of BH_2_ to BH_4_ is comparable between the sexes (males: 0.41 ± 0.14; females: 0.43 ± 0.05). Therefore, it is unlikely that there are sex differences in BH_2_ competition for NOS binding. Consistent with our hypothesis, tempol increased BH_4_ levels, although BH_2_ levels were also increased in both male and female SHR following tempol treatment and total biopterin increased in male SHR. While we cannot account for the mechanism mediating the increase in BH_2_, it is likely due to the fact that systemic tempol treatment does not abolish all oxidative stress in the renal IM. As a result, the increase in BH_2_ in male SHR may reflect an increase in total biopterin levels in an oxidative stress environment. In contrast, BH_2_ levels in female SHR increase in the absence of a change in total biopterins, suggesting a sex-specific effect of oxidative stress on biopterins. Future studies will be designed to examine this sex difference.

To further assess the role of endogenous BH_4_ in modulating NOS activity in male SHR and explore the potential therapeutic role of BH_4_ treatment, male SHR were treated with exogenous BH_4_ for 1 week. Supplementation with BH_4_ decreased dependency of NOS enzymatic activity on excess BH_4_, further supporting the hypothesis that a BH_4_ deficiency in male SHR contributes to lower levels of NOS activity compared with females. Interestingly, total NOS activity was decreased by 7 days of BH_4_ treatment. Future studies will investigate the mechanism driving the decrease in NOS activity; however, the corresponding increase in BH_2_ with BH_4_ supplementation may compete with BH_4_ for binding to NOS thereby resulting in a decrease in total NOS activity. BH_4_ supplementation also decreased BP in male SHR, although it is unlikely that a decrease in BP alone was responsible for the decreased NOS activity as we have previously published that decreasing BP in male SHR with hydrochlorothiazide and reserpine has no effect on renal NOS activity [15]. Indeed, the finding that BH_4_ supplementation reduces BP despite reducing NOS activity in the IM suggests that inner medullary NOS is not a significant determinant of BP in male SHR. Instead, our result is consistent with previous reports in male SHR indicating that BH_4_ reduces testosterone synthesis thereby reducing BP [37]. In addition to decreasing BP, BH_4_ has also been shown to ameliorate cardiac hypertrophy and diastolic dysfunction in male SHR [35]. Genomic analysis of *GCH1*, which encodes GTPCH1, in humans found a sex-specific risk of hypertension in patients with a specific polymorphism of the *GCH1* gene, with females having significantly higher BP than males and lower NO production [38]. Given this finding in human patients, along with the currently reported sex difference in BH_4_ levels, it is important to consider sex as a biological variable in future studies assessing the therapeutic potential of BH_4_ in hypertension.

In conclusion, although it is well established that there is a sexual dimorphism in NO bioavailability [11–14], the molecular mechanisms responsible are still being investigated. NOS enzymatic activity is tightly regulated by numerous biochemical pathways [39], including the availability of the cofactor BH_4_. We found sex differences in BH_4_ in the renal IM of SHR which corresponds to sex differences in NOS activity and NO production. Further studies are needed to better elucidate the potential role of BH_4_ as a therapeutic agent in both sexes.

## Clinical perspectives

- Premenopausal females have a lower incidence of hypertension than age-matched males, however the mechanisms contributing to this are not well understood.
- This is the first study to report a sex difference in BH_4_ levels in a rodent model of hypertension. BH_4_ is an essential cofactor for the production of NO, a potent vasodilator that plays an essential role in BP regulation.
- Targetting BH_4_ may serve as a novel therapeutic pathway for the treatment of hypertension in both sexes.

## Abbreviations

3-NT: 3-nitrotyrosine
BH_2_: dihydrobiopterin
BH_4_: tetrahydrobiopterin
BP: blood pressure
GTPCH1: GTP cyclohydrolase-1
IM: inner medulla
NOS: nitric oxide synthase
SHR: spontaneously hypertensive rat
TBS-T: TBS, 0.1% Tween 20
